# Smoking identified as preferred mode of opioid safe supply use; investigating correlates of smoking preference through a 2021 cross-sectional study in British Columbia

**DOI:** 10.1186/s13011-023-00515-4

**Published:** 2023-05-16

**Authors:** Ariba Kamal, Max Ferguson, Jessica C Xavier, Lisa Liu, Brittany Graham, Kurt Lock, Jane A. Buxton

**Affiliations:** 1 Public Health Agency of Canada, Ottawa, Ontario Canada; 2 British Columbia Centre for Disease Control, Vancouver, British Columbia Canada; 3 School of Population and Public Health, University of British Columbia, Vancouver, British Columbia Canada

**Keywords:** Opioids, Harm reduction, Safe supply, Mode of consumption, Smoking, People who use drugs (PWUD)

## Abstract

**Background:**

The increasing number of illicit drug toxicity deaths in British Columbia (BC) has led to calls for a regulated (pharmaceutical grade) supply of substances (“safe supply”). In order to inform safe supply recommendations, we aimed to identify why people currently smoke opioids and assess the preferred mode of consumption if people who use opioids were provided with opioid safe supply.

**Methods:**

The BC Harm Reduction Client Survey (HRCS) is an annual survey that gathers information about people who use drugs' (PWUD) substance use characteristic with the goal of contributing to evidence-based policy. This study utilized data from the 2021 HRCS. The outcome variable was “prefer smoking opioid safe supply” (‘yes/no’). Explanatory variables included participants’ demographics, drug use, and overdose characteristics. Bivariate and hierarchical multivariable logistic regressions were conducted to identify factors associated with the outcome.

**Results:**

Of 282 total participants who indicated a preference for a mode of consumption for opioid safe supply, 62.4% preferred a smokable option and 19.9% preferred to inject if provided with opioid safe supply. Variables significantly associated with the outcome (preferred smoking) included: being 19-29 years old (AOR=5.95, CI =1.93 – 18.31) compared to >50 years old, having witnessed an overdose in the last 6 months (AOR=2.26, CI=1.20 – 4.28), having smoked opioids in the last 3 days (AOR=6.35, CI=2.98 – 13.53) and having a preference to smoke stimulants safe supply (AOR=5.04, CI=2.53 – 10.07).

**Conclusion:**

We found that over half of participants prefer smokable options when accessing opioid safe supply. Currently in BC, there are limited smokable opioid safe supply options as alternatives to the toxic street supply. To reduce overdose deaths, safe supply options should be expanded to accommodate PWUD that prefer smoking opioids.

**Supplementary Information:**

The online version contains supplementary material available at 10.1186/s13011-023-00515-4.

## Background

The drug toxicity crisis is an ongoing public health concern in North America. The US Centers for Disease Control and Prevention estimated 100,306 drug overdose deaths occurred during a 12-month period ending in April 2021. This represents a 28.5% increase from the same period the year prior suggesting that the crisis has worsened in the US [1].

Approximately, 5,368 opioid toxicity deaths occurred in Canada in 2021 (January to September) averaging to 20 deaths per day [2, 3]. British Columbia (BC), Alberta, and Ontario are three provinces in Canada that accounted for 88% of all opioid toxicity deaths in 2021 (January to September) [3].

In response to rising numbers of overdose deaths, British Columbia declared a public health emergency in 2016 [4]. Illicit drug toxicity deaths account for more unnatural deaths in the province than homicides, suicides, motor vehicle incidents, drowning, and fire-related deaths combined [5]. In 2021 alone, there were 2,236 suspected illicit drug toxicity deaths in BC, averaging 6 deaths per day. This represents a 26% increase in deaths compared to 2020 and marks the highest number of deaths to ever have been recorded in a year in the province [6].

The high rate of illicit drug toxicity deaths observed is due to an unregulated, unpredictable, and toxic street supply of drugs. Around 2016, fentanyl began to saturate the illicit supply of opioids and many were unknowingly using this potent opioid [7]. Fentanyl, an opioid 50 to 100 times more potent than morphine, was detected (alone or as part of polysubstance) in over 85% of the illicit drug toxicity deaths in 2021 [8].

In March 2020, a second public health emergency was declared due to the COVID-19 pandemic [9]. As a result, public health measures enacted to create physical distancing also impacted people who use drugs (PWUD) access to harm reduction and healthcare services. Furthermore, social isolation was suspected to have increased the number of people using drugs alone and border closures led to disruptions in the illicit drug trade and supply [5]. Consequently, PWUD experienced a market with an increasingly unpredictable toxic drug supply [10]. Together, these changes led to an increased risk of overdose and an increase in the number of illicit drug toxicity deaths from the time of the declaration of the COVID-19 pandemic to present.

Safe^1^ supply refers to “a legal and regulated supply of drugs with mind/body altering properties that traditionally have been accessible only through the illicit drug market [11]. The objective is to separate PWUD from a toxic drug supply by providing access to a supply of pharmaceutical grade drugs of known concentration and constituents [12]. Opioid Agonist Therapy (OAT) involves the prescription of an opioid medication to prevent withdrawal and reduce cravings for opioid drugs, but does not provide non-medicinal properties [13]. Whereas safe supply aims to provide access to substances that provide non-medicinal and/or euphoric properties that many people seek from the illicit drug supply [14].

A new policy directive on prescribed safer supply in BC was developed in 2021, which led to the development and expansion of safe supply programs in the province including the SAFER Initiative – Safer Alternative for Emergency Response [14, 15]. While these developments signal support for safe supply programs, the policy directive offers limited options in terms of types of drugs, dosages, and modes of consumption. Hence, for some PWUD the new policy has not met the criteria of an ideal safer supply model, and therefore, did not entirely remove the incentive to access the illicit drug supply [5, 16].

The BC Coroners Service found that the mode of consumption associated with illicit drug toxicity deaths in BC has changed over time with the street drug supply becoming increasingly toxic due to greater potency of fentanyl, its analogues and other substance [5]. Injection as the mode of consumption decreased from 39% in 2016 to 19% in 2020, whereas smoking increased from 31% to 56% during the same time period [17]. Literature suggests that fear of needles and bloodborne illnesses as well as perceived high risk of overdose with injecting are amongst some of the many reasons that motivate PWUD to choose smoking as their mode of consumption [18]. Although smoking has been found as the most common mode of illicit drug consumption amongst decedents, the current safe supply programs do not offer many safe supply options that are smokable [5, 16].

The goal of safe supply is to reduce the risks associated with using drugs from the highly toxic illicit supply. As safe supply programs are designed and implemented, it is important that we consider opioid preferences, preferred mode of consumption, and the unique realities and needs of different groups of PWUD. This will allow us to create programs that are acceptable, accessible, and appropriate to the population they aim to benefit. A research study by Parent et al. revealed that smoking was the commonest mode of opioid consumption amongst the participants that visited harm reduction sites in BC in 2019 with two-thirds of the participants reporting smoking as their mode of consumption [18]. To our knowledge, to date, there has been limited research on PWUDs’ preference for the different modes of consumption when using opioid safe supply.

The aim of this study is to inform safe supply programs to better meet the needs of PWUD by 1) exploring reasons why PWUD currently smoke opioids, 2) investigating their preference for smoking and non-smoking modes of consumption if provided with a pharmaceutical grade opioid safe supply, and 3) identifying factors associated with smoking opioid safe supply.

## Materials and methods

### Research procedures

The STROBE Cross-sectional Checklist^2^ was utilized to strengthen our study reporting [19].

**Fig. 1:**
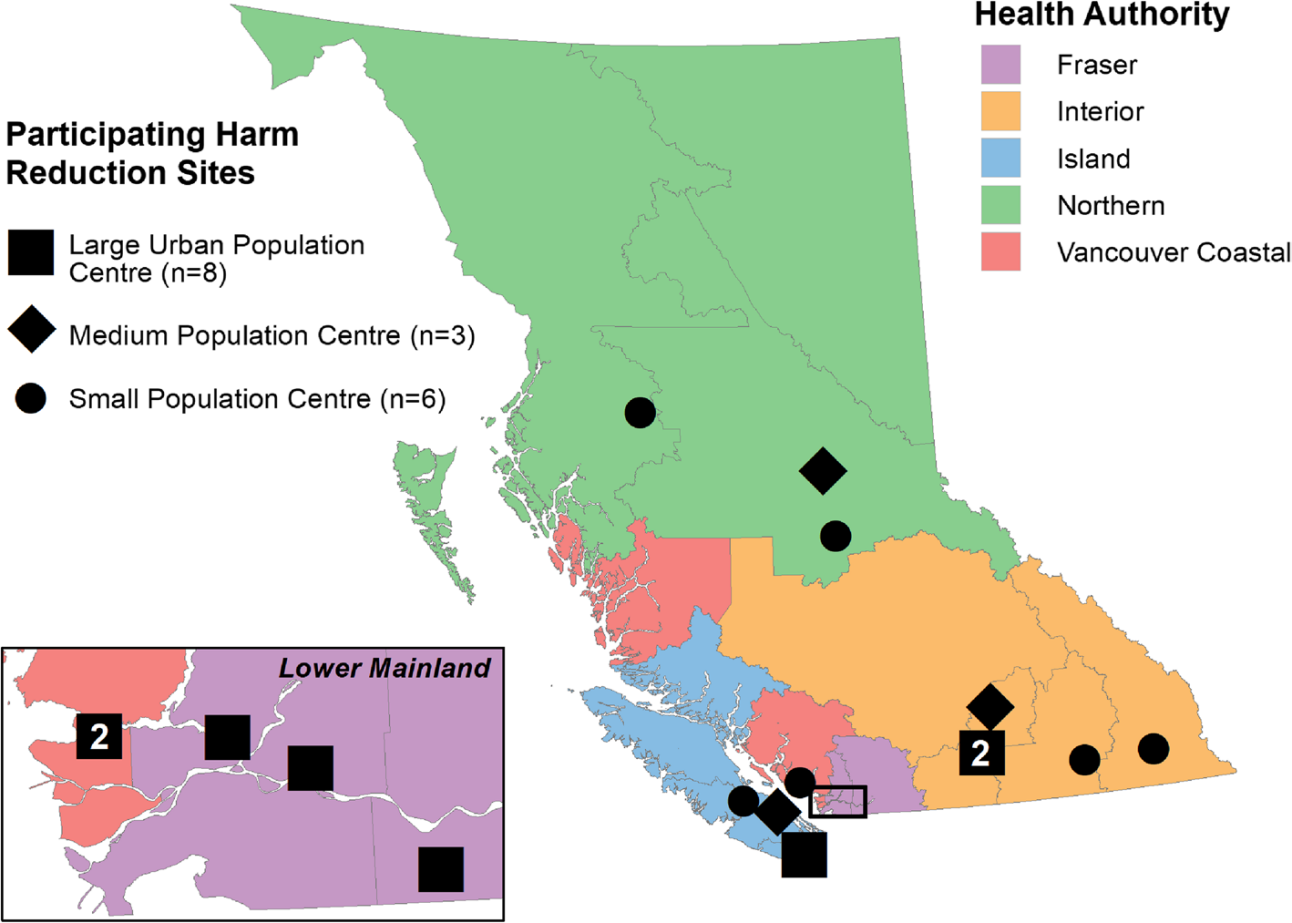
2021 Harm Reduction Client Survey site map

The cross-sectional Harm Reduction Client Survey^3^ (HRCS) was piloted in BC in 2012 and has since been used in 2013, 2014, 2015, 2018, and 2019 by the BC Centre for Disease Control (BCCDC) [20]. The HRCS aims to gather information about substance use characteristics, access and barriers to harm reduction services and resources, with the goal of contributing to evidence-based policy and programming. Clients at harm reduction supply distribution sites across BC were invited to complete the survey (see Fig. 1). Participants were recruited via convenience sampling by trained staff and volunteers based on their willingness and consent to participate. This in-person survey was administered at harm reduction supply distribution sites with the assistance of site staff and peer workers. More information about the HRCS data collection methods can be found in previous publications [21–24].

Inclusion criteria for participants were: being 19 years or older, self-reported use of any illicit substances other than or in addition to cannabis in the past six months, and the ability to provide verbal informed consent. The questionnaire took approximately 20 minutes to complete. Upon completing the survey, participants received $15 CAD for participating and the sites received $5 for every participant that was recruited. Data entry and analysis occurred at the BCCDC.

### Study measures

Our study’s outcome variable was “prefer smoking opioid safe supply” which was dichotomized to ‘yes – prefer smoking opioid safe supply/no – do not prefer smoking opioid safe supply’. The composite variable was created by combining participants who had answered “yes” to preferring to smoke diacetylmorphine (heroin), fentanyl, hydromorphone, morphine, and oxycodone (e.g., OxyContin, OxyNeo) as alternatives to street down (unknown opioids) or opioids. Participants that had answered “yes” to preferring to snort, inject, swallow or use other mode of consumption when using diacetylmorphine (heroin), fentanyl, hydromorphone, morphine, oxycodone (e.g., OxyContin, OxyNeo) were aggregated under the “do no prefer smoking opioid safe supply” category.

Explanatory variables included participant demographics, drug use, and overdose characteristics.

#### Demographic factors

Demographic factors included urbanicity^4^ of survey administration sites (large urban population centre, medium urban population centre, small population centre), age category (19–29, 30–39, 40–49, ≥ 50, unknown), gender (cis woman, cis man, trans, gender expansive (trans man, trans woman, gender non-conforming, other specified gender), unknown), sexual orientation^5^ (LGBTQ, non-LGBTQ, unknown), health authority (Fraser Health, Interior Health, Island Health, Northern Health, Vancouver Coastal Health), Indigenous identity (Indigenous (including First Nations, Métis and Inuit) , non-Indigenous, unknown), housing status^6^ (stably housed, not stably housed, unknown), employment status^7^ (employed, not employed, unknown), having a disability^8^ (yes, no, unknown).

#### Overdose characteristics

Overdose characteristics included whether the participant currently perceived themselves to be at risk of overdose (yes, no, unknown), whether participant had witnessed an opioid overdose in the last six months (yes, no, unknown), and whether participant had experienced an opioid overdose in the last six months (yes, no, unknown).

#### Drug use characteristics

Drug use characteristics included how frequently the participant used drugs alone (ever^9^, never, unknown), whether the participant used overdose prevention site (yes, no, unknown), if participants were prescribed opioid agonist therapy (yes, no, unknown), how frequently did the participant use drugs in the last month (everyday, a few times a week, a few times a month, did not use drugs, unknown), drugs reported used in the last three days (methadone, buprenorphine, hydromorphone, oxycodone, morphine, fentanyl &/or diacetylmorphine (heroin)^10^ , Xanax, other benzos, crystal meth, cocaine, crack, MDMA, other stimulants, cannabis, tobacco, alcohol), smoked opioid in last three days (yes, no), smoked opioid/down in the last month (yes, no, unknown), smoked/inhaled any drug in the last six months (yes, no, unknown), and prefer to smoke stimulants safe supply (yes, no). Multiple questions related to time frame of smoking opioids (three days and last month) were included in the survey. Due to the relevance of these measures, we included all of them in model building and assessed for collinearity by looking at variance inflation factor.

### Analytic sample

**Fig. 2:**
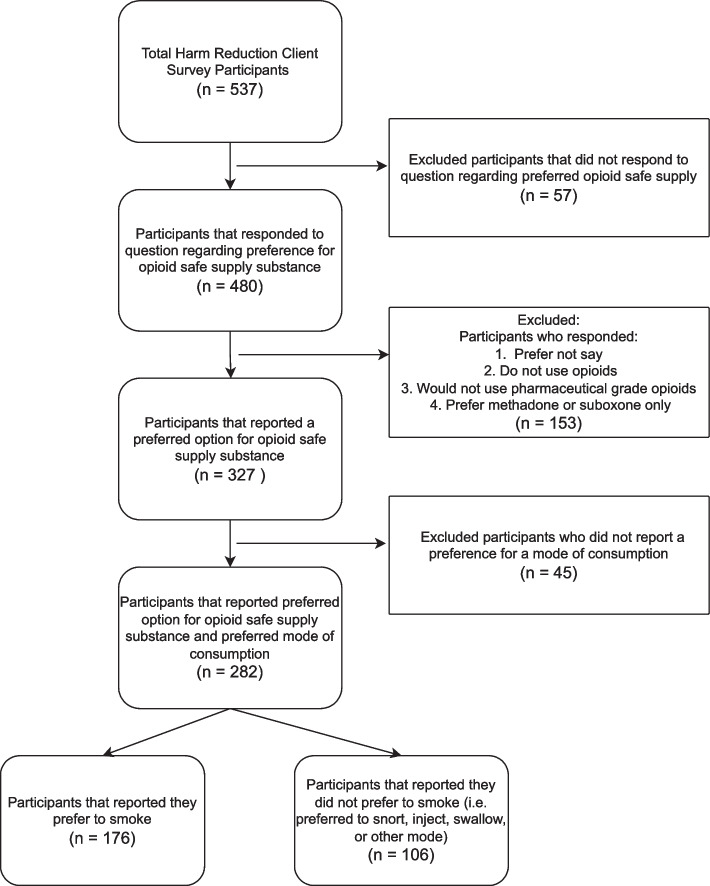
Flow chart of Analytic Sample

The harm reduction client survey inquired about participants’ preference for the various opioid safe supply as well as their preferred mode of consumption (Question 14 – see Appendix 1). Only respondents who reported a preference for an opioid safe supply and those who indicated a preference for a mode of consumption for opioid safe supply were included in the analytic sample as shown in Fig. 2. Descriptive statistics, bivariate regression as well as multivariable regression analyses were conducted using this analytic sample.

An “Unknown” category was developed for explanatory variables. This included missing, invalid, “prefer not to say” or “I don’t know” responses.

### Data analysis

A frequency table of the reasons why participants currently prefer to smoke opioid over other methods was created (Question 20b of survey – see Appendix 1). From the participants that had responded “yes” to having smoked opioids/down in the past month (n = 232), 161 provided a reason for preferring to smoke.

Additionally, frequency tables were created for all variables of interest and all explanatory variables were also stratified by “smoking opioid safe supply preference”. Bivariate logistic regression analyses were conducted to estimate the association between the explanatory variables and preference for smoking opioid safe supply [29]. Fisher’s Exact tests were conducted for variables with more than two categories [30]. Alpha, or the significant level is set to 0.05 (5%).

Cochrane-Armitage test was carried out to evaluate the trend in preference to smoke opioid safe supply by increasing age for participants whose age was known [31].

For multivariable regression, a 3-block hierarchical modelling approach was utilized [32]. Models were constructed to investigate the influence of demographics (block one), overdose characteristics (block two) as well as drug use characteristics (block three) on the odds of preferring to smoke opioid safe supply over other modes of consumption. To build the model for each block, bivariate logistic regression for each explanatory variable was performed and variables with p value < 0.25 were included in the model building process in line with purposeful selection [29]. Variables with any level with p-value under 0.25 in bivariate regression were evaluated for model inclusion using a backwards selection approach based on achieving the lowest the value of Akaike’s information criteria (AIC) [33]. Variables that were deemed conceptually important were retained in the model (i.e., gender). The final selected model included the variables age, gender, health authority, having witnessed overdose, frequency of drug use in the last six months, having smoked opioids in the last three days, preferring to smoke stimulants safe supply, using cannabis/hash, and alcohol in the last three days. Although fentanyl &/or diacetylmorphine, other benzos, and crystal meth/methamphetamine were significant in the bivariate regression, they were not retained in the adjusted model.

To demonstrate the relative contribution of each block to model fit, the likelihood ratio ${R}^{2}$ was calculated after the inclusion of each subsequent block [34]. Models were also compared with each other using the likelihood ratio test where each model was compared to the model in the previous step [35]. Variance inflation factor was used to assess collinearity [36].

Unadjusted odds ratios (UOR), adjusted odds ratio (AOR), 95% confidence intervals (CI), and p-values were reported. Analysis was performed using R Statistical Software (Version 4.1.2) [37]. Alpha, or the significant level is set to 0.05 (5%). Significance at p ≤ 0.05 level is demarcated in Tables 2 and 4.

## Results

### Current reasons for smoking

Amongst the respondents who currently smoke opioids over other methods, it was found that 47.6% of the total responses demonstrated that participants preferred to smoke opioids due to safety reasons, 26.6% preferred the effect and practice of smoking opioids and 25.8% do not inject as shown in Table 1. Within the 47.6% that preferred to smoke opioids due to safety reasons, 14% believed that they were less likely to overdose, 12.6% felt they were less likely to get blood borne diseases, 11.2% felt they were less likely to get other infections followed by 9.7% that believed that by smoking opioids they were better able to control dosage.

**Table 1:** Reasons participants gave for preferring to smoke opioids/down compared to other methods

**Reasons for smoking opioids**^**A**^		**N (%)**^**B**^	**Overall %**
Safety reasons	Less likely to overdose	49 (14.0%)	47.6%
	Less likely to get blood borne disease e.g. HIV/HCV	44 (12.6%)	
	Less likely to get other infections e.g. abscess	39 (11.2%)	
	Better able to control dosage	34 (9.7%)	
Effect and practice of smoking	Prefer the effects from smoking	45 (12.9%)	26.6%
	Smoking is more social	25 (7.2%)	
	Able to smoke together with stimulants e.g. crystal meth	20 (5.7%)	
	Prefer the practice of smoking	3 (0.9%)	
Do not Inject	Do not like injecting	40 (11.5%)	25.8%
	Never injected	27 (7.7%)	
	Can no longer inject/cannot find vein	23 (6.6%)	

Note: ^A^The reasons were provided by 161 participants of the 232 who  responded 'yes' to having smoked any opioid/down in the past month

^B^ Total # of responses = 349, % is of all responses

Question asked to select all that apply, hence, these categories are not mutually exclusive

Other specified reasons were re-categorized into existing categories where appropriate or a new category was developed

### Demographics of the study sample

Table 2 shows demographics and drug use characteristics for the 282 participants who indicated that they would be interested in opioid safe supply. A large proportion of the participants were cis men (64.2%), were 40 years and older (58.1%), identified as non-LGBTQ (79.8%), identified as non-Indigenous (51.4%), lived in medium and small population centres (73.4%), were currently unemployed (75.9%), were currently stably housed (56%), and reported having a disability (81.2%). Descriptive analyses of Indigenous (First Nations and Métis), non-Indigenous identity and unknown are presented in Table 2. Pan-Indigenous variables might be unable to show important differences experienced by Indigenous peoples in Canada, therefore, descriptive data on First Nations and Métis participants is provided (no people who reported a preference for mode of consumption for opioid safe supply identified as Inuit in the analytic sample). In terms of overdose characteristics, a majority of the respondents had witnessed an opioid overdose in the last 6 months (70.9%), and 32% had experienced an opioid overdose in the last 6 months. In terms of drug use characteristics, 86.2% of the respondents had used drugs alone, 52.1% had OAT prescribed in the last 6 months, 73% had smoked opioid in the last three days of having taken the survey, and 53.5% had shown a preference to smoke stimulants if provided with a safe supply of stimulants.

**Table 2:** Characteristics of the 2021 HRCS participants stratified by preference for smoking opioid safe supply

**Characteristics**	**Mode of Consumption Preference**		**Total (n=282)**	**Bivariate Regression**	**Fisher’s Exact Test**
	**Prefer smoking opioid safe supply** **(n=176)** n (row %)	**Did not prefer smoking opioid safe supply** **(n=106)** n (row %)	**n (column %)**	**P-value**	**P-value**
**Age Category**					
≤29	29 (85.3%)	5 (14.7%)	34 (12.1%)	<0.01***	<0.01***
30-39	52 (69.3%)	23 (30.7%)	75 (26.6%)	0.01**	
40-49	45 (60.8%)	29 (39.2%)	74 (26.2%)	0.13	
≥50	44 (48.9%)	46 (51.1%)	90 (31.9%)	Reference	
Unknown	6 (66.7%)	3 (33.3%)	9 (3.2%)	0.32	
**Gender**					
Cis man	118 (65.2%)	63 (34.8%)	181 (64.1%)	Reference	0.32
Cis woman	52 (58.4%)	37 (41.6%)	89 (31.6%)	0.28	
Transgender and gender expansive	2 (40.0%)	3 (60.0%)	5 (1.8%)	0.26	
Unknown	4 (57.1%)	3 (42.9%)	7 (2.5%)	0.66	
**Sexual Orientation**					
LGBTQ	24 (61.5%)	15 (38.5%)	39 (13.8%)	Reference	0.99
Non-LGBTQ	141 (62.7%)	84 (37.3%)	225 (79.8%)	0.89	
Unknown	11 (61.1%)	7 (38.9%)	18 (6.4%)	0.98	
**Urbanicity**					
Large urban population centre	47 (62.7%)	28 (37.3%)	75 (26.6%)	Reference	0.22
Medium population centre	82 (67.2%)	40 (32.8%)	122 (43.3%)	0.52	
Small population centre	47 (55.3%)	38 (44.7%)	85 (30.1%)	0.35	
**Health Authority**					
Fraser Health	31 (79.5%)	8 (20.5%)	39 (13.8%)	Reference	<0.01***
Interior Health	37 (56.1%)	29 (43.9%)	66 (23.4%)	0.02*	
Island Health	48 (60.8%)	31 (39.2%)	79 (28.0%)	0.05*	
Northern Health	50 (78.1%)	14 (21.9%)	64 (22.7%)	0.87	
Vancouver Coastal Health	10 (29.4%)	24 (70.6%)	34 (12.1%)	<0.01***	
**Indigenous Identity**^**A**^					
Indigenous^B^	71 (59.7%)	48 (40.3%)	119 (42.2%)	Reference	0.31
First Nations	50 (59.5%)	34 (40.5%)	84 (70.6%)	-	
Métis	21 (60.0%)	14 (40.0%)	35 (29.4%)	-	
Non-Indigenous	96 (66.2%)	49 (33.8%)	145 (51.4%)	0.27	
Unknown	9 (50.0%)	9 (50.0%)	18 (6.4%)	0.44	
**Currently Employed**^**C**^					
Employed	27 (55.1%)	22 (44.9%)	49 (17.4%)	Reference	0.25
Unemployed	139 (65.0%)	75 (35.0%)	214 (75.9%)	0.20	
Unknown	10 (52.6%)	9 (47.4%)	19 (6.7%)	0.85	
**Currently Stably Housed**^**D**^					
Yes	92 (58.2%)	66 (41.8%)	158 (56.0%)	0.09	0.10
No	81 (68.1%)	38 (31.9%)	119 (42.2%)	Reference	
Unknown	3 (60.0%)	2 (40.0%)	5 (1.8%)	0.71	
**Have a Disability**					
Yes	141 (61.6%)	88 (38.4%)	229 (81.2%)	0.24	0.34
No	15 (75.0%)	5 (25.0%)	20 (7.1%)	Reference	
Unknown	20 (60.6%)	13 (39.4%)	33 (11.7%)	0.29	
**Have a perceived risk of opioid overdose**					
Yes	68 (61.3%)	43 (38.7%)	111 (39.4%)	0.58	0.60
No	86 (64.7%)	47 (35.3%)	133 (47.2%)	Reference	
Unknown	22 (57.9%)	16 (42.1%)	38 (13.4%)	0.45	
**Have witnessed an opioid overdose in the last 6 months**					
Yes	133 (66.6%)	67 (33.5%)	200 (70.9%)	<0.01**	<0.01**
No	30 (46.9%)	34 (53.1%)	64 (22.7%)	Reference	
Unknown	13 (72.2%)	5 (27.8%)	18 (6.4%)	0.06	
**Have experienced opioid overdose in the last 6 months**					
Yes	61 (64.2%)	34 (35.8%)	95 (32.0%)	0.68	0.69
No	103 (61.7%)	64 (38.3%)	167 (61.8%)	Reference	
Unknown	12 (60.0%)	8 (40.0%)	20 (6.2%)	0.88	
**Use Drugs Alone**					
Ever	157 (64.6%)	86 (35.4%)	243 (86.2%)	0.08	0.11
Never	15 (48.4%)	16 (51.6%)	31 (11.0%)	Reference	
Unknown	4 (50.0%)	4 (50.0%)	8 (2.8%)	0.94	
**Used Overdose Prevention Site**					
Yes	54 (58.1%)	39 (41.9%)	93 (33.0%)	0.38	0.43
No	108 (63.5%)	62 (36.5%)	170 (60.3%)	Reference	
Unknown	14 (73.7%)	5 (26.3%)	19 (6.7%)	0.38	
**Opioid Agonist Therapy Prescribed**					
Yes	94 (63.9%)	53 (36.1%)	147 (52.1%)	0.80	0.89
No	63 (62.4%)	38 (37.6%)	101 (35.8%)	Reference	
Unknown	19 (55.9%)	15 (44.1%)	34 (12.1%)	0.50	
**Frequency of drug use in last month**					
Every day	146 (68.9%)	66 (31.1%)	212 (75.2%)	Reference	<0.01***
A few times a week	14 (35.0%)	26 (65.0%)	40 (14.2%)	<0.001***	
A few times a month	4 (40.0%)	6 (60.0%)	10 (3.5%)	0.07	
Did not use	1 (100.0%)	0 (0.0%)	1 (0.4%)	0.99	
Unknown	11 (57.9%)	8 (42.1%)	19 (6.7%)	0.32	
**Drugs used in the last 3 days**					
**Opioids**					
Fentanyl &/or Diacetylmorphine (Heroin)					
Yes	163 (66.5%)	82 (33.5%)	245 (86.9%)	<0.01***	-
No	13 (35.1%)	24 (64.9%)	37 (13.1%)	Reference	
Hydromorphone (e.g. Dilaudid)					
Yes	69 (63.9%)	39 (36.1%)	108 (38.3%)	0.69	-
No	107 (61.5%)	67 (38.5%)	174 (61.7%)	Reference	
Morphine (e.g. Kadian or M-Eslon)					
Yes	45 (58.4%)	32 (41.6%)	77 (27.3%)	0.40	-
No	131 (63.9%)	74 (36.1%)	205 (72.7%)	Reference	
Oxycodone (e.g. OxyContin, OxyNeo)					
Yes	14 (58.3%)	10 (41.7%)	24 (8.5%)	0.67	-
No	162 (62.8%)	96 (37.2%)	258 (91.5%)	Reference	
Methadone (Methadose/Metadol)					
Yes	65 (61.3%)	41 (38.7%)	106 (37.6%)	0.77	-
No	111 (63.1%)	65 (36.9%)	176 (62.4%)	Reference	
Buprenorphine/Naloxone (Suboxone or Sublocade)					
Yes	11 (64.7%)	6 (35.3%)	17 (6.0%)	0.84	-
No	165 (62.3%)	100 (37.7%)	265 (94.0%)	Reference	
Xanax					
Yes	15 (71.4%)	6 (28.6%)	21 (7.4%)	0.38	-
No	161 (61.7%)	100 (38.3%)	261 (92.6%)	Reference	
Other Benzos (e.g. Ativan/Valium)					
Yes	61 (74.4%)	21 (25.6%)	82 (29.1%)	<0.01**	-
No	115 (57.5%)	85 (42.5%)	200 (70.9%)	Reference	
**Stimulants**					
Crystal Meth/Methamphetamine					
Yes	156 (69.0%)	70 (31.0%)	226 (80.1%)	<0.01***	-
No	20 (35.7%)	36 (64.3%)	56 (19.9%)	Reference	
Cocaine (powder)					
Yes	32 (59.3%)	22 (40.7%)	54 (19.1%)	0.60	-
No	144 (63.2%)	84 (36.8%)	228 (80.9%)	Reference	
Crack					
Yes	50 (66.7%)	25 (33.3%)	75 (26.6%)	0.37	-
No	126 (60.9%)	81 (39.1%)	207 (73.4%)	Reference	
MDMA/Ecstasy					
Yes	12 (80.0%)	3 (20.0%)	15 (5.3%)	0.16	-
No	164 (61.4%)	103 (38.6%)	267 (94.7%)	Reference	
Other stimulant (e.g. Ritalin/Adderall)					
Yes	17 (63.0%)	10 (37.0%)	27 (9.6%)	0.95	-
No	159 (62.4%)	96 (37.6%)	255 (90.4%)	Reference	
**Other/legal substances**					
Cannabis/Hash					
Yes	93 (69.4%)	41 (30.6%)	134 (47.5%)	0.05*	-
No	83 (56.1%)	65 (43.9%)	148 (52.5%)	Reference	
Tobacco (cigarettes)					
Yes	155 (64.3%)	86 (35.7%)	241 (85.5%)	0.11	-
No	21 (51.2%)	20 (48.8%)	41 (14.5%)	Reference	
Alcohol					
Yes	78 (69.6%)	34 (30.4%)	112 (39.7%)	0.05*	-
No	98 (57.6%)	72 (42.4%)	170 (60.3%)	Reference	
**Preference for opioid safe supply**					
Diacetylmorphine (Heroin)					
Yes	120 (77.4%)	35 (22.6%)	155 (55.0%)	<0.01***	-
No	56 (44.1%)	71 (55.9%)	127 (45.0%)	Reference	
Fentanyl (liquid)					
Yes	19 (55.9%)	15 (44.1%)	34 (12.1%)	0.40	-
No	157 (63.3%)	91 (36.7%)	248 (87.9%)	Reference	
Fentanyl (patch)					
Yes	18 (50%)	18 (50%)	36 (12.8%)	0.10	-
No	158 (64.2%)	88 (35.8%)	246 (87.2%)	Reference	
Fentanyl (powder)					
Yes	61 (82.4%)	13 (17.6%)	74 (26.2%)	<0.01***	-
No	115 (55.3%)	93 (44.7%)	208 (73.8%)	Reference	
Hydromorphone (injectable)					
Yes	10 (50.0%)	10 (50.0%)	20 (7.1%)	0.24	-
No	166 (63.4%)	96 (36.6%)	262 (92.9%)	Reference	
Hydromorphone (tablet e.g. Dilaudid)					
Yes	14 (50.0%)	14 (50.0%)	28 (9.9%)	0.16	-
No	162 (63.8%)	92 (36.2%)	254 (90.1%)	Reference	
Morphine (capsule/tablet e.g. Kadian/M-Eslon)					
Yes	13 (44.8%)	16 (55.2%)	29 (10.3%)	0.05*	-
No	163 (64.4%)	90 (35.6%)	253 (89.7%)	Reference	
Morphine (injectable)					
Yes	9 (34.6%)	17 (65.4%)	26 (9.2%)	0.01**	-
No	167 (65.2%)	89 (34.8%)	256 (90.8%)	Reference	
Oxycodone (e.g., OxyContin, OxyNeo)					
Yes	10 (38.5%)	16 (61.5%)	26 (9.2%)	0.01**	-
No	166 (64.8%)	90 (35.2%)	256 (90.8%)	Reference	
Methadone (Methadose/Metadol)					
Yes	15 (62.5%)	9 (37.5%)	24 (8.5%)	0.99	-
No	161 (62.4%)	97 (37.6%)	258 (91.5%)	Reference	
Buprenorphine/Naloxone (Suboxone)					
Yes	6 (85.7%)	1 (14.3%)	7 (2.5%)	0.23	-
No	170 (61.8%)	105 (38.2%)	275 (97.5%)	Reference	
Other					
Yes	10 (50.0%)	10 (50.0%)	20 (7.1%)	0.24	-
No	166 (63.4%)	96 (36.6%)	262 (92.9%)	Reference	
**Smoked opioid in last 3 days**					
Yes	153 (74.3%)	53 (25.7%)	206 (73.0%)	<0.01***	-
No	23 (30.3%)	53 (69.7%)	76 (27.0%)	Reference	
**Prefer to smoke stimulants safe supply**					
Yes	117 (77.5%)	34 (22.5%)	151 (53.5%)	<0.01***	-
No	59 (45.0%)	72 (55.0%)	131 (46.5%)	Reference	

^A^ The pan-Indigenous identity variable (whether someone reported being Indigenous or not) was used in the regression analyses to increase power to provide more meaningful information on associations with Indigenous identity. Data on First Nations and Métis participants are provided in our descriptives in realization that pan-Indigenous variables might not show important differences that may be experienced by Indigenous peoples in British Columbia

^B^ No one who identified as Inuit was included in the analytic sample

^C^ Employed included full-time work, part-time work, and paid volunteer

^D^ Stably housed was defined as living in private residence alone, living in private residence with someone else, and in another residence (living in hotels, motels, or social/supportive housing). Not stably housed was defined as being homeless, having no fixed address, couch surfing, or living in a shelter

^*^ Significant at 0.05

^**^ Significant at 0.01

^***^ Significant at 0.001

### Preference for the mode of consumption

Among the 282 participants that showed a preference for a mode of consumption for opioid safe supply, 73% indicated that they smoked opioids in the last three days and 62.4% indicated that they would prefer a smokable option of their preferred opioid safe supply. Although smoking was the preferred mode of consumption for opioid safe supply, 19.9% preferred to inject the opioids and 11.7% preferred to swallow the opioids, as shown in Table 3. Due to the nature of the question, these categories are not mutually exclusive.

**Table 3:** Preferred mode of consumption of opioid safe supply (*n*=282)

**Mode of Consumption**	**N (%)**
Smoking	176 (62.4%)
Injecting	56 (19.9%)
Swallowing	33 (11.7%)
Snorting	9 (3.2%)
Other	8 (2.8%)

The stratification of the study variables by mode of consumption preference (preference to smoke opioid safe supply) can be seen in Table 2. The prevalence for preference for smoking opioid safe supply was 65.2% amongst cis men whereas it was 58.4% amongst cis women. Preference for smoking opioid safe supply was more common amongst those who identified as non-LGBTQ (62.7%), were 29 years or younger (85.3%), were living in medium population centres (67.2%), were currently unemployed (65%), were not stably housed (68.1%), had witnessed an opioid overdose in the last 6 months (66.6%), reported using drugs alone occasionally, often or always (64.6%), and those that reported smoking opioids in the last three days (74.3%). The Cochran-Armitage test for trend between age (where age was known) and preference to smoke opioid safe supply was significant (*p* <0.001).

### Variables associated with preference for smoking opioid safe supply

Based on the bivariate regression, preference for smoking opioid safe supply significantly differed by age, health authority, having witnessed an opioid overdose in the last 6 months, frequency of using drugs alone, frequency of drug use in the last month, drugs used in the last three days (diacetylmorphine (heroin) & fentanyl, other benzos, crystal meth, cannabis/hash, tobacco, and alcohol), smoking opioids in last three days, and a preference for a smokable safe supply of stimulants.

Table 4 shows the hierarchical model findings. The regression equation is significant in block one on demographics, ${R}^{2}$= 0.12, *p*<0.001. Results indicate that participants who were 29 years of age or younger had 5.95 times the odds of preferring to smoke opioid safe supply compared to participants 50 years or older (AOR=5.95, 95%CI =1.93–18.31).

**Table 4:** Adjusted odds ratios and 95% confidence intervals for factors associated with preference for smoking opioid safe supply (*n*=282)

Smoking Opioid Safe Supply				
	**Simple Bivariate OR (95% CI)**	**Block 1 (Demographics)** **AOR (95% CI)**	**Block 2 (OD Characteristics)** **AOR (95% CI)**	**Block 3 (Drug Use Characteristics)** **AOR (95% CI)**
**Demographic Characteristics**				
**Age Category**				
≤29	6.06 (2.15 – 17.05)**	5.95 (1.93 – 18.31)***	5.90 (1.85 – 18.82)***	4.26 (0.96 – 18.90)
30-39	2.36 (1.24 – 4.49)**	2.51 (1.24 – 5.09)*	2.48 (1.21 – 5.10)**	1.59 (0.67 – 3.75)
40-49	1.62 (0.87 – 3.03)	1.53 (0.76 – 3.07)	1.43 (0.70 – 2.91)	1.56 (0.67 – 3.63)
≥50	—	—	—	—
Unknown	2.09 (0.49 – 8.88)	2.26 (0.46 –11.04)	2.17 (0.45 – 10.46)	5.00 (0.77 – 32.26)
**Gender**				
Cis man	—	—	—	—
Cis woman	0.75 (0.45 – 1.26)	0.68 (0.39 – 1.21)	0.68 (0.38 – 1.21)	0.87 (0.42 - 1.77)
Transgender & gender expansive	0.36 (0.06 – 2.19)	1.26 (0.16 – 9.67)	1.39 (0.16 – 11.87)	3.91 (0.25 – 60.01)
Unknown	0.71 (0.15 – 3.28)	1.08 (0.20 – 5.69)	0.89 (0.17 – 4.72)	1.22 (0.17 – 8.75)
**Health Authority**				
Fraser Health	—	—	—	—
Interior Health	0.33 (0.13 – 0.82)*	0.27 (0.11 – 0.71)**	0.31 (0.12 – 0.80)*	0.37 (0.12 – 1.11)
Island Health	0.40 (0.16 – 0.98)*	0.43 (0.17 – 1.09)	0.52 (0.20 – 1.34)	0.74 (0.25 – 2.24)
Northern Health	0.92 (0.35 – 2.45)	0.68 (0.24 – 1.87)	0.73 (0.26 – 2.04)	1.00 (0.31 – 3.21)
Vancouver Coastal Health	0.11 (0.04 – 0.31)***	0.10 (0.03 – 0.31)***	0.10 (0.03 – 0.32)***	0.05 (0.01 – 0.22)
**Overdose Characteristics**				
***Have witnessed an opioid overdose in the last 6 months***				
Yes	2.25 (1.27 – 3.99)**		2.26 (1.20 – 4.28)**	1.19 (0.53 – 2.66)
No	—		—	—
Unknown	2.95 (0.94 – 9.23)		1.78 (0.52 – 6.16)	0.72 (0.16 – 3.33)
**Drug Use Characteristics**				
***Frequency of drug use in the last month***				
Everyday	—			—
A few times a week	0.24 (0.12 – 0.50)***			0.16 (0.06 – 0.44)***
A few times a month	0.30 (0.08 – 1.10)			0.24 (0.05 – 1.17)
Did not use drugs	9.58exp^5^ (0.00 – Inf)			1.98exp8 (0.00 – inf)
Unknown	0.62 (0.24 – 1.62)			0.93 (0.26 – 3.34)
***Smoked Opioid last 3 days***				
Yes	6.65 (3.72 – 11.89)***			6.35 (2.98 – 13.53)***
No	—			—
***Prefer smoking stimulants safe*** **supply**				
Yes	4.20 (2.51 – 7.02)***			5.04 (2.53 – 10.07)***
No	—			—
***Drugs Used in the last 3 days Cannabis/Hash***				
Yes	1.78 (1.09 – 2.90)*			2.72 (1.36 – 5.44)**
No	—			—
***Alcohol***				
Yes	1.69 (1.02 - 2.79)*			1.95 (0.97 – 3.91)
No	—			—
LR Pseudo - ***R***^***2***^		0.12***	0.14*	0.35***
Pseudo – ***R***^***2***^ - change		0.12	0.02	0.21

*Abbreviations*: *AOR* adjusted odds ratio, *CI* confidence interval

^*^ Significant at 0.05

^**^ Significant at 0.01

^***^ Significant at 0.001

Including overdose characteristics in block two significantly improved the model fit, ${R}^{2}$ = 0.14, *p*<0.05. Those who had witnessed an overdose in the last 6 months had 2.26 times the odds of preferring smoking opioid safe supply over other modes of consumption in comparison to those who did not witness an opioid overdose in the last 6 months (AOR=2.26, 95%CI=1.20–4.28).

The addition of drug use characteristics in block three further improved the model fit, ${R}^{2}$= 0.35, *p*<0.001. Participants who had smoked opioids in the last three days had 6.35 times the odds of preferring to smoke opioid safe supply (AOR=6.35, 95%CI=2.98–13.53) than those that did not smoke opioids in the last three days. Participants that preferred smoking stimulants safe supply had 5.04 times the odds of preferring to smoke opioid safe supply than those who did not prefer smoking stimulants safe supply (AOR=5.04, 95%CI=2.53-10.07). Those who used cannabis/hash in the last three days had 2.72 times the odds of preferring to smoke opioid safe supply than those who did not use cannabis/hash (AOR=2.72, 95% CI=1.36-5.44). Additionally, those who used alcohol in the last 3 days had 1.95 times the odds of preferring to smoke opioid safe supply than those who did not use alcohol (AOR=1.95, 95%CI=0.97-3.91).

It was found that the variance inflation factor were all under 4 so within acceptable limits, therefore collinearity was not a concern in the model [36].

## Discussion

Our study investigated the prevalence of different preferred modes of consumption for opioid safe supply and identified factors associated with the preference for smoking opioid safe supply (the most frequently chosen mode of consumption) among people who use drugs who completed the 2021 HRCS.

A large proportion of participants reported smoking opioids in the last three days (73% of participants). In comparison, a smaller proportion of the same participants showed a preference to smoke if provided with opioid safe supply (62.4% of participants). Our findings also show that participants who witnessed an opioid overdose in the last six months had higher odds of preferring to smoke opioid safe supply. When asked why participants prefer to smoke their opioids, many of them indicated that they felt less at risk of overdosing from smoking than other modes of consumption. This is in line with a study conducted in Northern Ireland showing that a common perception amongst PWUD is that smoking substances reduces ones’ risk of overdose in comparison to injecting [38]. Given this perception of smoking as a safer use tactic and concerns around overdose due to a highly toxic drug supply, a high percentage of PWUD may be choosing to primarily smoke their street supply of opioids to try to reduce their risk of overdose. However, if provided with opioid safe supply, they may opt for other modes of consumption in place of smoking because they may be less concerned about unpredictable potencies and contaminants. This finding points to the importance of gaining a nuanced understanding of the reasons behind peoples’ substance use patterns and preferences to inform safe supply programs in the short term and, in the long term, to anticipate how peoples’ safe supply preferences and needs may shift as they are provided access to a regulated supply of a range of substances.

Provincial initiatives have been undertaken by the province of BC to address the public health concern of illicit drug toxicity deaths. OAT was expanded with the goal of addressing opioid withdrawal symptoms and reducing cravings for opioids. However, in BC, 12-month retention rates for OAT are 7.9%-18.1% depending on the medication prescribed, showing that that while OAT is a valuable tool to address some PWUD’s needs and objectives (e.g. those interested in managing withdrawal symptoms), it is not appropriate for all [39]. The Risk-Mitigation Guidance (RMG) was launched in March 2020 allowing the prescribing of pharmaceutical alternatives to PWUD to reduce overdose deaths and to limit the transmission of COVID-19 [10]. RMG permitted the prescribing of opioids, stimulants, and benzodiazepines. The opioids offered by RMG included hydromorphone and M-Eslon which were available in tablet form [10, 40]. Multiple sources have indicated that the substances and the forms available for consumption under RMG need to be expanded to accommodate the diverse needs and preferences of PWUD [5, 41]. The prescribed safer supply directive was another provincial initiative that was launched in BC in July 2021 and allowed for the availability of legal and regulated versions of some drugs that are normally accessible through the illicit drug market only. The SAFER initiative operating in Victoria is a result of this initiative and offers three fentanyl products including, fentanyl patches, fentanyl buccal tablets (Fentora) tablets, and sufentanil injections [16]. Injectable OAT (iOAT) is another form of safe supply that can prevent withdrawal amongst PWUD as well as provide non-medical effects similar to those of drugs acquired from the illegal drug market. Prescription diacetylmorphine (heroin) and hydromorphone due to their limited availability are offered through iOAT to a small number of patients [42]. Furthermore, tablet iOAT is also a form of safe supply which offers pharmaceutical-grade hydromorphone tablets [43, 44]. These various models are important as they are operationalizing safe supply directives with the common goal of providing a regulated supply of substances and reducing illicit drug toxicity deaths in the province. However, none of these models offer smokable forms of opioids and are therefore unable to accommodate those who have a preference for smoking opioid safe supply.

When developing and expanding safe supply options in BC, it is important to consider that there is an overwhelming preference for using smokable forms of opioids and a lack of smokable opioid safe supply, which may contribute to PWUD resorting to other modes of consumption such as injecting or continuing to rely on the illegal supply for smokable substances. Injecting drugs comes with its own risk including risks of blood borne diseases, risks of injection related injuries like abscesses, and vein collapse and bruising – risks that some PWUD may want to avoid. Table 1 shows the top few reasons why participants currently prefer to smoke opioids over other modes of consumption in the last month, which illustrate participants wish to reduce their own risks of harms including: a perception that one is less likely to overdose from smoking, smoking being associated with a reduced risk of infection or blood borne disease, and a preference to not inject. It is therefore important to offer safe supply programs that offer smokable opioid options to meet the needs of PWUD, a heterogeneous group with differing preferences and conditions.

Our findings also show that those aged 29 or younger had higher odds of preferring smoking as a mode of consumption if offered opioid safe supply compared to those 50 and older. This finding is consistent with a BC study that found that PWUD younger than 30 were more likely to prefer smoking opioids in comparison to those that were 50 and over [18]. The BC Coroner Service report showed that illicit drug toxicity deaths are the leading cause of death amongst the age group of 19–39-year-olds [45]. Given this preference for smoking opioids among young PWUD and the high rates of illicit drug toxicity deaths in this age group, smokable forms of safe supply are necessary to meet the needs of this sub-population of PWUD and reduce young peoples’ reliance on the toxic street supply.

Smoking was the most common mode of consumption found amongst people who died of illicit drug toxicity between August 2017 and July 2021, and recent evidence suggests that PWUD are primarily choosing to smoke over other modes of consumption (e.g., inject, swallow, snort, other) [17, 18]. Although there is a growing number of supervised consumption and overdose prevention service sites in BC, it has been found that less than a third (13) of the 42 locations offer inhalation services [46]. The overall goal of safe supply programs is to provide regulated drugs to people to reduce the risk of overdose; however, existing programs have yet to include more options that could, at minimum, improve overdose response and reversal among PWUD who commonly smoke their substances. Until viable alternatives become available to the public, setting up more inhalation sites across BC could reduce overdose deaths among people who smoke illicit drugs [47]. Increasing access to supervised inhalation sites could also improve awareness of health and social services in PWUD who primarily smoke their drugs, much like supervised injection sites have served as an avenue to connect people who inject drugs to additional services and resources [47]. Low-barrier supervised inhalation sites may also increase access to alternatives to the toxic illicit supply by serving as an entry point for PWUD to gain awareness of, and access to, smokable safe supply options.

Our findings suggest that participants who drank alcohol or used cannabis or hash in the last 3 days had higher odds of preferring to smoke opioids safe supply. More research is required to examine this association and potential contributing factors.

Presently, PWUDs are interested in accessing safe supply programs but smokable safe supply options are limited. Excluding certain types of opioids and modes of consumption increases the risk of overdose for particular sub-groups of PWUD, as they are likely to continue to rely on the toxic street supply to meet their needs [41]. Offering smokable options for opioids safe supply should be considered for those who will be deterred from accessing safe supply programs limited to injectable or oral routes of administration. From a health equity perspective, it is paramount that safe supply programs be designed and implemented to ensure that they are accessible to as many PWUD as possible, and especially PWUD in rural and remote locations who face more barriers to harm reduction and overdose response services as well as medical and social service supports. Consultation with PWUD is essential for the successful development of programs that meet the needs of PWUDs. Therefore, exploratory qualitative research is recommended to gain a nuanced understanding from PWUD about needs and intentions informing peoples' preferences for safe supply programs in terms of substances, modes of consumption and models offered.

## Limitations

The study was based on a client survey; therefore, it employed the use of self-reported data which may have introduced bias. Furthermore, we are unable to infer causal relationships due to the cross-sectional nature of the study. Moreover, the questions in the survey ask specifically about drugs and demographics (i.e., employment, and housing status), therefore, the presence of social desirability bias would need to be considered. It should be noted that the participants in this study were individuals who had accessed harm reduction sites and for that reason the finding of this study cannot be generalized to all people who use drugs in the province of British Columbia especially those who are less connected to services and may not prefer to disclose their substance use. Due to the multiple tests performed in the study, the potential increase for Type I error among the many tests presented is another limitation.

## Conclusion

Our findings show that if people who use opioids were provided with opioid safe supply, more than half would prefer to smoke opioids. The findings highlight important correlates such as age, witnessing opioid overdose, and having smoked opioids in the last 3 days. By taking these findings into consideration, safe supply program implementation can be improved to meet the needs of PWUD by including drugs that are smokable. In order to better understand the experiences and preferences of PWUD, studies employing qualitative methods are recommended.

## Supplementary Information

**Additional file 1.** 

## Data Availability

The datasets used and/or analysed during the current study are available from the corresponding author upon reasonable request.

## Abbreviations

BC: British Columbia
HRCS: Harm Reduction Client Survey
PWUD: People Who Use Drugs
OAT: Opioid Agonist Therapy
SAFER: Safer Alternative for Emergency Response
PEEP: Professionals for Ethical Engagement of Peers
UOR: Unadjusted Odds Ratio
AOR: Adjusted Odds Ratio
CI: Confidence Interval
iOAT: Injectable Opioid Agonist Therapy

## Footnotes

1 We use the term safe as this was present in the wording of the questions in the HRCS and often used by people who use drugs [11]. However, the authors acknowledge that using a regulated supply of opioids is not safe, and safer may be the preferred term.

2 STROBE Cross-sectional checklist was obtained online (https://www.strobe-statement.org/checklists/)

3 The Harm Reduction Client Survey can be accessed on the BCCDC website (http://www.bccdc.ca/resource-gallery/Documents/Statistics%20and%20Research/Statistics%20and%20Reports/Overdose/2021%20BC%20Harm%20Reduction%20Client%20Survey.pdf )

4 Urbanicity was determined using 2021 Statistics Canada census (https://geosuite.statcan.gc.ca/geosuite/en/index) and the 2016 population centre and rural area classification [25, 26]. Size of population and population density were taken into consideration when classifying.

5 We categorized Two-Spirit identity separately from Western gender and sexual orientation identities [27]. There was insufficient sample size to report on Two-Spirit findings in this analysis.

6 Stably housed was defined as living in private residence alone, living in private residence with someone else, and in another residence (living in hotels, motels, or social/supportive housing). Not stably housed was defined as currently being homeless, having no fixed address, couch surfing, or living in a shelter.

7 Employment status was classified as employed (paid volunteer, part-time work, full-time work), not employed and unknown.

8 Disability included sensory, physical, cognitive and mental health-related and anything the participants identified as a disability.

9 Used drugs alone Ever includes those who answered “occasionally”, “often”, or “always”.

10 Fentanyl and diacetylmorphine (heroin) are combined after taking into consideration that survey takers respond taking heroin, but drug checking reveals that samples contain fentanyl [28].

## References

[1] Drug Overdose Deaths in the US Top 100,000 Annually [Internet]. National Centre for Health Statistics; 2021 [cited 2022 Mar 20]. Available from: https://www.cdc.gov/nchs/pressroom/nchs_press_releases/2021/20211117.htm.

[2] Mattson CL, Tanz LJ, Quinn K, Kariisa M, Patel P, Davis NL. Trends and Geographic Patterns in Drug and Synthetic Opioid Overdose Deaths — United States, 2013–2019. MMWR. Morbidity and Mortality Weekly Report [Internet]. 2021 [cited 2022 Jul 11];70. Available from: https://www.cdc.gov/mmwr/volumes/70/wr/mm7006a4.htm. 10.15585/mmwr.mm7006a4 PMC7877587 33571180

[3] Opioid- and Stimulant-related Harms in Canada Published: (June 2022) [Internet]. Public Health Agency of Canada: Special Advisory Committee on the Epidemic of Opioid Overdoses; 2022. Available from: https://health-infobase.canada.ca/substance-related-harms/opioids-stimulants.

[4] Health. Provincial health officer declares public health emergency | BC Gov News [Internet]. Government of British Columbia; 2016 [cited 2022 Jul 11]. Available from: https://news.gov.bc.ca/releases/2016HLTH0026-000568.

[5] BC Coroners Service Death Review Panel: A Review of Illicit Drug Toxicity Deaths [Internet]. BC Coroners Service; 2022 [cited 2022 Mar 30]. Available from: https://www2.gov.bc.ca/assets/gov/birth-adoption-death-marriage-and-divorce/deaths/coroners-service/death-review-panel/review_of_illicit_drug_toxicity_deaths_2022.pdf.

[6] Illicit Drug Toxicity Deaths in BC [Internet]. Ministry of Public Safety & Solicitor General; 2022 Jun [cited 2022 Jul 11]. Available from: https://www2.gov.bc.ca/assets/gov/birth-adoption-death-marriage-and-divorce/deaths/coroners-service/statistical/illicit-drug.pdf.

[7] Kuo M. The BC Public Health Opioid Overdose Emergency [Internet]. BC Centre for Disease Control; 2017 Mar. Available from: http://www.bccdc.ca/resource-gallery/Documents/Educational%20Materials/Epid/Other/Public%20Surveillance%20Report_2017_03_17.pdf.

[8] Illicit Drug Toxicity Type of Drug Data to April 30, 2022 [Internet]. British Columbia: Ministry of Public Safety and Solicitor General; 2022 Apr [cited 2022 Jul 20]. Available from: https://www2.gov.bc.ca/assets/gov/birth-adoption-death-marriage-and-divorce/deaths/coroners-service/statistical/illicit-drug-type.pdf.

[9] Henry B. Provincial health officer notice [Internet]. Office of the Provincial Health Officer, Ministry of Health; 2020. Available from: https://www2.gov.bc.ca/assets/gov/health/about-bc-s-health-care-system/office-of-the-provincial-health-officer/reports-publications/pho-regional-event-notice.pdf.

[10] Ahamad K, Bach P, Brar R, Chow N, Coll N, Compton M, et al. Risk Mitigation in the Context of Dual Public Health Emergencies [Internet]. BC Centre on Substance Use; 2022 Mar [cited 2022 Mar 30]. Available from: https://www.bccsu.ca/wp-content/uploads/2020/04/Risk-Mitigation-in-the-Context-of-Dual-Public-Health-Emergencies-v1.5.pdf.

[11] Safe Supply Concept Document [Internet]. Canadian Association of People who Use Drugs; 2019 [cited 2022 Mar 20]. Available from: https://vancouver.ca/files/cov/capud-safe-supply-concept-document.pdf.

[12] Access to Prescribed Safer Supply in British Columbia: Policy Direction [Internet]. Ministry of Mental Health and Addictions Ministry of Health; 2021 [cited 2022 Mar 20]. Available from: https://www2.gov.bc.ca/assets/gov/overdose-awareness/prescribed_safer_supply_in_bc.pdf.

[13] Opioid Agonist Therapy [Internet]. Centre for Addiction and Mental Health; 2016 [cited 2022 Jul 11]. Available from: https://www.camh.ca/-/media/files/oat-info-for-clients.pdf.

[14] B.C. Introduces New Prescribed Safer Supply Policy, a Canadian First | BC Gov News [Internet]. Ministry of Mental Health and Addictions; 2021 [cited 2022 Jul 11]. Available from: https://news.gov.bc.ca/releases/2021MMHA0035-001375.

[15] PHS Community Services Society. PHS to Deliver Multi-Year Safe Supply Program | PHS Community Services Society [Internet]. PHS. 2021 [cited 2022 Jul 11]. Available from: https://www.phs.ca/phs-to-deliver-multi-year-safe-supply-program/.

[16] Ranger C, Hobbs H, Cameron F, Stuart H, McCall J Sullivan G, et al. Co/Lab Practice Brief: Implementing the Victoria SAFER Initiative. [Internet] Canadian Institute for Substance Use Research; 2021 [cited 2022 Nov 12]. Available from: https://www.uvic.ca/research/centres/cisur/assets/docs/colab/practice-brief-safer.pdf.

[17] Illicit Drug Toxicity Deaths in BC Knowledge Update: Mode of Consumption [Internet]. British Columbia: Ministry of Public Safety & Solicitor General; 2022 Feb. Available from: https://www2.gov.bc.ca/assets/gov/birth-adoption-death-marriage-and-divorce/deaths/coroners-service/statistical/mode-of-consumption.pdf.

[18] S Parent K Papamihali B Graham BJA Examining Prevalence and Correlates of Smoking Opioids in British Columbia: Opioids Are More Often Smoked than Injected. Substance Abuse Treatment, Prevention, and Policy [Internet]. 2021 Oct 18 [cited 2022 Jul 11];16(1):79 Available from: 10.1186/s13011-021-00414-6. 10.1186/s13011-021-00414-6 PMC8522853 34663374

[19] STROBE Checklist: Cohort, Case-Control, and Cross-Sectional Studies (Combined) [Internet]. STROBE - Strengthening the Reporting of Observational Studies in Epidemiology. STROBE; [cited 2022 Jul 11]. Available from: https://www.strobe-statement.org/.

[20] Harm reduction client survey [Internet]. BC Centre for Disease Control; [cited 2022 Jul 11]. Available from: http://www.bccdc.ca/health-professionals/data-reports/harm-reduction-client-survey.

[21] Moustaqim-Barrette A, Papamihali K, Crabtree A, Graham B, Karamouzian M, Buxton JA. Correlates of Take-Home Naloxone Kit Possession among People Who Use Drugs in British Columbia: A Cross-Sectional Analysis. Drug and Alcohol Dependence [Internet]. 2019 Dec 1 [cited 2022 Jul 11];205:107609. Available from: https://www.sciencedirect.com/science/article/pii/S0376871619303862. 10.1016/j.drugalcdep.2019.107609 31654839

[22] M Kuo A Shamsian D Tzemis BJAA Drug U Survey among Clients of Harm Reduction Sites across British Columbia, Canada, 2012. Harm Reduction Journal [Internet]. 2014 Apr 27 [cited 2022 Jul 11];11(1):13 Available from: 10.1186/1477-7517-11-13. 10.1186/1477-7517-11-13 PMC4016659 24766846

[23] Papamihali K, Yoon M, Graham B, Karamouzian M, Slaunwhite AK, Tsang V, et al. Convenience and Comfort: Reasons Reported for Using Drugs Alone among Clients of Harm Reduction Sites in British Columbia, Canada [Internet]. 2020 [cited 2022 Jul 11]. Available from: https://open.library.ubc.ca/soa/cIRcle/collections/facultyresearchandpublications/52383/items/1.0395074. 10.1186/s12954-020-00436-6 PMC7682134 33228676

[24] Karamouzian M, Papamihali K, Graham B, Crabtree A, Mill C, Kuo M, et al. Known Fentanyl Use among Clients of Harm Reduction Sites in British Columbia, Canada. International Journal of Drug Policy [Internet]. 2020 Mar 1 [cited 2022 Jul 11];77:102665. Available from: https://www.sciencedirect.com/science/article/pii/S0955395920300062. 10.1016/j.drugpo.2020.102665 31962283

[25] Population Centre and Rural Area Classification 2016 [Internet]. [cited 2022 Jul 11]. Available from: https://www.statcan.gc.ca/en/subjects/standard/pcrac/2016/introduction.

[26] Government of Canada. Geosuite [Internet]. Statistics Canada; 2016 [cited 2022 Jul 11]. Available from: https://geosuite.statcan.gc.ca/geosuite/en/index.

[27] Pruden H, Salway T. Meet the Methods series: “What and who is Two-Spirit?” in Health Research [Internet]. Two-Spirit Dry Lab. 2020 [cited 2022 Sep 17]. Available from: https://twospiritdrylab.ca/what-and-who-is-two-spirit/.

[28] Payer DE, Young, MM, Maloney-Hall B, Mill C, Leclerc P, Buxton J, the Canadian Community Epidemiology Network on Drug Use, & the National Drug Checking Working Group. Adulterants, contaminants and co-occurring substances in drugs on the illegal market in Canada: An analysis of data from drug seizures, drug checking and urine toxicology [Internet]. Canadian Centre on Substance Use and Addiction. 2020 [cited 2022 Sep 17]. Available from: https://www.ccsa.ca/sites/default/files/2020-04/CCSA-CCENDU-Adulterants-Contaminants-Co-occurring-Substances-in-Drugs-Canada-Report-2020-en.pdf.

[29] Zhang Z. Model Building Strategy for Logistic Regression: Purposeful Selection. Ann Transl Med [Internet]. 2016 Mar [cited 2022 Jul 11];4(6):111. Available from: https://www.ncbi.nlm.nih.gov/pmc/articles/PMC4828741/. 10.21037/atm.2016.02.15 PMC4828741 27127764

[30] Kim HY. Statistical notes for clinical researchers: Chi-squared test and Fisher’s exact test. Restor Dent Endod [Internet]. 2017 May [cited 2022 Dec 31];42(2):152–5. Available from: https://www.ncbi.nlm.nih.gov/pmc/articles/PMC5426219/. 10.5395/rde.2017.42.2.152 PMC5426219 28503482

[31] Hirji KF, Tang ML. A Comparison of Tests for Trend. Communications in Statistics - Theory and Methods [Internet]. 1998 Jan 1 [cited 2022 Jul 11];27(4):943–63. Available from: 10.1080/03610929808832137.

[32] Kim B. Hierarchical Linear Regression | University of Virginia Library Research Data Services + Sciences [Internet]. [cited 2022 Jul 11]. Available from: https://data.library.virginia.edu/hierarchical-linear-regression/.

[33] Akaike H. Akaike’s Information Criterion. In: Lovric M, editor. International Encyclopedia of Statistical Science [Internet]. Berlin, Heidelberg: Springer; 2011 [cited 2022 Jul 11]. p. 25–25. Available from: 10.1007/978-3-642-04898-2_110.

[34] Cohen J, Cohen P, West S, Aiken L. Applied Multiple Regression/Correlation Analysis for the Behavioral Sciences [Internet]. 2003 [cited 2022 Jul 11]. Available from: https://www.routledge.com/Applied-Multiple-RegressionCorrelation-Analysis-for-the-Behavioral-Sciences/Cohen-Cohen-West-Aiken/p/book/9780805822236.

[35] Hosmer DW, Lemeshow S, Sturdivant RX. Model-Building Strategies and Methods for Logistic Regression [Internet]. 1st ed. Wiley; 2013 [cited 2022 Jul 11]. (Applied Logistic Regression, Third Edition). Available from: 10.1002/9781118548387.

[36] Collinearity diagnostics [Internet]. [cited 2022 Jul 11]. Available from: https://cran.r-project.org/web/packages/olsrr/vignettes/regression_diagnostics.html.

[37] The R Project for Statistical Computing [Internet]. R. [cited 2022 Jul 11]. Available from: https://www.r-project.org/.

[38] Harris J, Shorter GW, Davidson G, Best P. Risk Perception, Changing Social Context, and Norms Prevent Transition to Regular Injection among People Who Smoke Heroin. Drug and Alcohol Dependence [Internet]. 2020 Mar 1 [cited 2022 Jul 11];208:107878. Available from: https://www.sciencedirect.com/science/article/pii/S0376871620300430. 10.1016/j.drugalcdep.2020.107878 32014646

[39] Kurz M, Min JE, Dale LM, Nosyk B. Assessing the Determinants of Completing OAT Induction and Long-Term Retention: A Population-Based Study in British Columbia, Canada. Journal of Substance Abuse Treatment [Internet]. 2022 Feb 1 [cited 2022 Jul 11];133:108647. Available from: https://www.sciencedirect.com/science/article/pii/S0740547221003731. 10.1016/j.jsat.2021.108647 PMC9833672 34740484

[40] Nosyk B, Slaunwhite A, Urbanoski K, Hongdilokkul N, Palis H, Lock K, et al. Evaluation of Risk Mitigation Measures for People with Substance Use Disorders to Address the Dual Public Health Crises of COVID-19 and Overdose in British Columbia: A Mixed-Method Study Protocol. BMJ Open [Internet]. 2021 Jun 1 [cited 2022 Jul 11];11(6):e048353. Available from: https://bmjopen.bmj.com/content/11/6/e048353. 10.1136/bmjopen-2020-048353 PMC8190984 34108170

[41] McMurchy D, Palmer H. Assessment of the Implementation of Safer Supply Pilot Projects. [Internet]. Health Canada; March 2022 [cited 2022 Nov 12]. Available from: https://www.nss-aps.ca/sites/default/files/resources/2022-03-safer_supply_preliminary_assessment_report_en_0.pdf.

[42] Klaire S, Sutherland C, Kerr T, Kennedy MC. A low-barrier, flexible safe supply program to prevent deaths from overdose. CMAJ. 2022 May 16;194(19):E674–6. Available from: https://www.cmaj.ca/content/194/19/E674. 10.1503/cmaj.211515 PMC9438736 35577374

[43] Ahamad K, Compton M, Dolman C, Fairbairn N, Foreman J, Gustafon R, et al. Guidance for Injectable Opioid Agonist Treatment for Opioid Use Disorder [Internet]. BC Centre on Substance Use; [cited 2022 Jul 11]. Available from: https://www.bccsu.ca/wp-content/uploads/2021/07/BC_iOAT_Guideline.pdf.

[44] Weng J, Fairbairn N, Sutherland C, Johnson C, Nolan S. Supervised Tablet Injectable Opioid Agonist Therapy (TiOAT): A Strategy to Address Safer Supply for Individuals With an Opioid Use Disorder? J Addict Med. 2022 Jun 1;16(3):258–60. Available from: https://pubmed.ncbi.nlm.nih.gov/34145188/. 10.1097/ADM.0000000000000887 34145188

[45] Top 15 Causes of Death [Internet]. BCCDC Mortality Context App. [cited 2022 Jul 11]. Available from: https://bccdc.shinyapps.io/Mortality_Context_ShinyApp/.

[46] Unregulated Drug Poisoning Emergency Dashboard [Internet]. Tableau Software. [cited 2022 Sep 17]. Available from: https://public.tableau.com/views/UnregulatedDrugPoisoningEmergencyDashboard/Introduction?%3Adisplay_static_image=y&%3AbootstrapWhenNotified=true&%3Aembed=true&%3Alanguage=en-US&:embed=y&:showVizHome=n&:apiID=host0#navType=0&navSrc=Parse.

[47] Bourque S, Pijl EM, Mason E, Manning J, Motz T. Supervised Inhalation Is an Important Part of Supervised Consumption Services. Can J Public Health [Internet]. 2019 Apr 1 [cited 2022 Jul 11];110(2):210–5. Available from: 10.17269/s41997-019-00180-w. 10.17269/s41997-019-00180-w PMC6964381 30725386

