# Supplementary material for: Smoking identified as preferred mode of opioid safe supply use; investigating correlates of smoking preference through a 2021 cross-sectional study in British Columbia
Source: Subst Abuse Treat Prev Policy. 2023 May 16;18:27. doi: 10.1186/s13011-023-00515-4 (PMC10186314; doi:10.1186/s13011-023-00515-4)
Supplement: Supplementary file 1 — Additional file 1. [file 13011_2023_515_MOESM1_ESM.pdf]

Place ID # Sticker Here

2020 Harm Reduction Client Survey

This survey is being conducted to help improve harm reduction services. No personal identifying information will be collected and your responses will be kept confidential. Your participation is voluntary and you are free to only answer the questions you are comfortable with. We will also be asking you about potential impacts of COVID-19 (corona virus) on accessing harm reduction and substance use services. You do not have to answer any questions, including questions about COVID-19 (corona virus), that may be triggering or may cause you discomfort. A "prefer not to say" option exists for every question. Not answering any question will not disqualify you from participating. Support services are available, and a list can be provided by the research coordinator. The survey will take roughly 20 minutes of your time and you will be asked to provide a urine sample. Please note that you can only complete the survey once.

To participate in this survey, you must have used an illegal drug within the last 6 months.

1. What is your current gender identity? (Select one)
☐ Woman ☐ Man ☐ Trans man ☐ Trans woman ☐ Two-Spirit
☐ Gender non-conforming ☐ Other, specify: ______________________ ☐ Prefer not to say

2. How old are you? ____________(years) ☐ Prefer not to say

3. Do you identify as any of the following? (Select one)
☐ First Nations ☐ Métis ☐ Inuit ☐ No, I don't identify as any of these ☐ Prefer not to say

4. Were you born in Canada (Select one)
☐ Yes ☐ No ☐ Prefer not to say

5. Were your parents born in Canada? (Select one)
☐ Yes ☐ No ☐ Prefer not to say

6. Which best describes your sexual orientation? (Select one)
☐ Gay ☐ Lesbian ☐ Straight ☐ Bisexual ☐ Queer ☐ Two-Spirit
☐ Other, specify ____________________ ☐ Prefer not to say

7. Which of the following options best describes where you currently live? (Select one)
☐ In a private residence, alone ☐ In a private residence, with someone else
☐ In another residence (hotels/motels, rooming houses, single room occupancy (SRO), social/supportive housing)
☐ In a shelter ☐ I have no regular place to stay (homeless, couch surf, no fixed address)
☐ Other, specify ______________________________ ☐ Prefer not to say

8. Are you currently employed? (Select all that apply)
☐ Yes, paid volunteer ☐ Yes, part -time ☐ Yes, full-time ☐ No ☐ Prefer not to say

9. Do you have any children in your care? (Select one)
☐ Yes ☐ No ☐ Prefer not to say

10. Do you identify as a person living with a disability related to any of the following? (Select one)
☐ Sensory (e.g. seeing, hearing) ☐ Physical (e.g. mobility, flexibility, dexterity, pain)
☐ Cognitive (e.g. learning, developmental, memory) ☐ Mental health-related
☐ Other, specify ________________________________ ☐ Prefer not to say

11. Do you have a cellphone? (Select one)
☐ Yes ☐ No ☐ Prefer not to say

**12. Did you use any of these in the *last 3 days*?** (Check off all that apply)		**a. If YES, how did you use it?** (Circle all that apply)					**b. If YES, did you have a prescription for it?** (Check off all that apply)
**Methadone (Methadose/Metadol)**	☐	Smoke	Snort	Inject	Swallow	Other	☐
**Buprenorphine/Naloxone (Suboxone or Sublocade)**	☐	Smoke	Snort	Inject	Swallow	Other	☐
**Hydromorphone (e.g. Dilaudid)**	☐	Smoke	Snort	Inject	Swallow	Other	☐
**Oxycodone (e.g., OxyContin, OxyNeo)**	☐	Smoke	Snort	Inject	Swallow	Other	☐
**Morphine (e.g. Kadian or M-Eslon)**	☐	Smoke	Snort	Inject	Swallow	Other	☐
**Heroin**	☐	Smoke	Snort	Inject	Swallow	Other	☐
**Fentanyl**	☐	Smoke	Snort	Inject	Swallow	Other	☐
**Xanax**	☐	Smoke	Snort	Inject	Swallow	Other	☐
**Other benzos (e.g. Ativan/Valium)**	☐	Smoke	Snort	Inject	Swallow	Other	☐
**Crystal Meth/Methamphetamine**	☐	Smoke	Snort	Inject	Swallow	Other	
**Cocaine (powder)**	☐	Smoke	Snort	Inject	Swallow	Other	
**Crack**	☐	Smoke	Snort	Inject	Swallow	Other	
**MDMA/Ecstasy**	☐	Smoke	Snort	Inject	Swallow	Other	
**Other stimulants (e.g. Ritalin/Adderall)**	☐	Smoke	Snort	Inject	Swallow	Other	☐
**Cannabis/Hash**	☐	Smoke			Swallow	Other	☐
**Tobacco (cigarettes)**	☐	Smoke			Chew	Other	
**Alcohol**	☐				Swallow	Other	
**Other 1, specify:** ______________	☐	Smoke	Snort	Inject	Swallow	Other	☐
**Other 2, specify:** ______________	☐	Smoke	Snort	Inject	Swallow	Other	☐

13. In the *last month*, how often did you use drugs by any mode (excluding cannabis, alcohol, or tobacco)? (Select one)
☐ Every day ☐ A few times a week ☐ A few times a month ☐ Did not use drugs ☐ Prefer not to say

**14. If you were prescribed a continuous supply of pharmaceutical grade down/opioids that is easy to access as an alternative to street opioids, which one would be your first choice?** (Check off ONE)		**b. If YES, how would you prefer to use the pharmaceutical grade down/opioids?** (Circle all that apply)				
**Methadone (Methadose/Metadol)**	☐	Smoke	Snort	Inject	Swallow	Other
**Buprenorphine/Naloxone (Suboxone)**	☐	Smoke	Snort	Inject	Swallow	Other
**Hydromorphone (injectable)**	☐	Smoke	Snort	Inject	Swallow	Other
**Hydromorphone (tablet, e.g. Dilaudid)**	☐	Smoke	Snort	Inject	Swallow	Other
**Morphine (capsule/tablet, e.g. Kadian/M-Eslon)**	☐	Smoke	Snort	Inject	Swallow	Other
**Morphine (injectable)**	☐	Smoke	Snort	Inject	Swallow	Other
**Oxycodone (e.g., OxyContin, OxyNeo)**	☐	Smoke	Snort	Inject	Swallow	Other
**Fentanyl (liquid)**	☐	Smoke	Snort	Inject	Swallow	Other
**Fentanyl (patch)**	☐	Smoke	Snort	Inject	Swallow	Other
**Fentanyl (powder)**	☐	Smoke	Snort	Inject	Swallow	Other
**Heroin/diacetylmorphine**	☐	Smoke	Snort	Inject	Swallow	Other
**Other, specify:** ______________________	☐	Smoke	Snort	Inject	Swallow	Other
I would not use pharmaceutical grade opioids	☐					
I do not use opioids/does not apply	☐					
Prefer not to say	☐					

**15. If you were prescribed a continuous supply of pharmaceutical grade stimulants that is easy to access as an alternative to street stimulants, which one would be your first choice?** (Check off ONE)		**b. If YES, how would you prefer to use the pharmaceutical grade stimulant?** (Circle all that apply)				
**Dextroamphetamine (Dexedrine)**	☐	Smoke	Snort	Inject	Swallow	Other
**Methylphenidate (Ritalin)**	☐	Smoke	Snort	Inject	Swallow	Other
**Crystal Meth/Methamphetamine (speed)**	☐	Smoke	Snort	Inject	Swallow	Other
**Cocaine (powder)**	☐	Smoke	Snort	Inject	Swallow	Other
**Crack**	☐	Smoke	Snort	Inject	Swallow	Other
**MDMA (Ecstasy)**	☐	Smoke	Snort	Inject	Swallow	Other
**Other, specify:** ______________________	☐	Smoke	Snort	Inject	Swallow	Other
I would not use pharmaceutical grade stimulants	☐					
I do not use stimulants/does not apply	☐					
Prefer not to say	☐					

16. Pandemic prescribing (sometimes called Risk Mitigation Guidance) allows physicians to prescribe some opioids, stimulants, and benzodiazepines so that people who use drugs can access safer drugs to prevent withdrawal and to allow physical distancing during the COVID-19 pandemic. Have you heard of pandemic prescribing? (Select one)

☐ Yes ☐ No *(skip to 17)* ☐ Prefer not to say *(skip to 17)*

a. Have you tried to obtain drugs through pandemic prescribing? (Select one)

☐ Yes, I tried and received a prescription ☐ Yes, I tried but physician would not prescribe *(skip to 17)*
☐ No, I have not tried but I want to *(skip to 17)* ☐ No, I have not tried and I do not want to *(skip to 17)*
☐ Other, specify: ______________________ ☐ Prefer not to say *(skip to 17)*

b. What drugs did you receive a prescription for? (Select all that apply)

☐ Opioids ☐ Stimulants ☐ Benzodiazepines ☐ Other, specify: __________ ☐ Prefer not to say

17. In the *last 6 months*, have you been prescribed Opioid Agonist Treatment (OAT)/Opioid Substitution Treatment (OST) (e.g. methadone, Suboxone (buprenorphine/naloxone), etc)? (Select one)

OAT/OST are drug therapies that counter opioid withdrawal symptoms and act as a substitute for the opioids you were previously taking

☐ Yes, I was prescribed OAT/OST ☐ No, but I use opioids and I'm interested in OAT/OST *(skip to 18)*
☐ No, I use opioids but I'm not interested *(skip to 18)* ☐ This does not apply to me, I don't use opioids *(skip to 18)*
☐ Other, specify: ______________________ ☐ Prefer not to say *(skip to 18)*

a. In the *last 6 months*, which OAT/OST did you get from the pharmacy? (Select all that apply)

☐ Methadone (Methadose) ☐ Buprenorphine/naloxone (Suboxone) ☐ Morphine (Kadian/M-eslon)
☐ Hydromorphone, pill form (Dilaudid) ☐ Hydromorphone, injectable(Dilaudid)
☐ Diacetylmorphine (heroin) ☐ None, I do not have a prescription *(skip to 18)*
☐ Other, specify: ______________________________ ☐ Prefer not to say

b. In the last 6 months, did you stop taking OAT/OST? (Select one)

☐ Yes ☐ No *(skip to 18)* ☐ Prefer not to say *(skip to 18)*

c. Why did you stop taking OAT/OST? (Select all that apply)

☐ Couldn't get to pharmacy during open hours ☐ Couldn't make clinic appointment time
☐ The OAT/OST treatment wasn't effective
☐ The dose of my OAT/OST was too low ☐ Switched treatment
☐ Clinic was too far away ☐ Clinic staff had negative attitude
☐ Pharmacy was too far away ☐ Pharmacy staff had a negative attitude
☐ I couldn't get the OAT/OST I wanted ☐ Worried about being exposed to COVID-19
☐ Other, specify: ______________________ ☐ Prefer not to say

18. What is your preferred method of using drugs? (Select one)

☐ Smoking/inhalation ☐ Snorting ☐ Injecting ☐ Swallowing
☐ It depends on substance, desired effect, setting, who I am with, etc. ☐ I do not have a preference
☐ Other, specify: ______________________________ ☐ Prefer not to say

19. How often do you use drugs alone? (Select one)
☐ Never ☐ Occasionally ☐ Often ☐ Always ☐ Prefer not to say

20. Have you smoked any opioids or down (e.g. fentanyl, heroin) in the past month? (Select one)
☐ Yes ☐ No *(skip to 21)* ☐ Prefer not to say *(skip to 21)*

a. **Do you prefer to smoke opioids/down over injecting and/or other methods of use?** (Select one)
☐ Yes ☐ No, I prefer other methods *(skip to 21)*
☐ No, I do not have a preference *(skip to 21)* ☐ Prefer not to say *(skip to 21)*

b. **Why do you prefer to smoke opioids/down compared to other methods?** (Select all that apply)
☐ Less likely to get a bloodborne disease, like HIV/hepatitis C, than from injecting
☐ Less likely to get other infections, like abscesses ☐ Can no longer inject/cannot find a vein
☐ I have never injected ☐ I don't like injecting ☐ Better able to control dose I am taking
☐ Less likely to overdose from smoking than other methods ☐ I prefer the effect from smoking
☐ Able to smoke together with stimulants (e.g., crystal meth) ☐ Smoking is more social
☐ Other, specify: ______________________ ☐ Prefer not to say

c. **Are you more likely to smoke opioids/down alone (by yourself), compared to other methods of using (e.g. snorting, injecting, swallowing, other)?** (Select one)
☐ Yes ☐ No *(skip to 21)* ☐ Prefer not to say *(skip to 21)*

d. **What are some of the reasons why you smoke opioids alone?** (Select all that apply)
☐ I am less likely to overdose while smoking opioids than other methods ☐ To be safe from others
☐ It's more convenient and comfortable to use at home ☐ I don't want to share
☐ I don't want others to know that I'm using drugs ☐ I don't have anyone else to use with
☐ Worried about being exposed to COVID-19 while using with others/at observed consumption sites
☐ Other, specify: ______________________ ☐ Prefer not to say

21. Have you used any of these drugs checking services or tools in the past 6 months? (Select all that apply)
☐ FTIR drug checking machine ☐ Mail-in drug checking ☐ Fentanyl test strips
☐ Benzo test strips ☐ Other, specify ______________________
☐ I have not used any of these services/tools ☐ Prefer not to say

a. **What has prevented you from using any drug checking services and/or tools?** (Select all that apply)
☐ I don't feel the need/want to use them ☐ Don't find them helpful
☐ I trust my source/dealer ☐ Don't want to give up drugs for drug checking
☐ Don't know where to find them ☐ No sites in my area ☐ Site was too far away
☐ Site not open when I needed it ☐ Site closed due to COVID-19 pandemic
☐ Concerned about confidentiality ☐ Worried about being exposed to COVID-19
☐ Other, specify______________________ ☐ Prefer not to say

22. If your drugs tested positive for fentanyl (before using), what would you do? (Select all that apply)
☐ Use less ☐ Use more slowly ☐ Would not use the drugs ☐ Continue using as usual
☐ Have someone check on me ☐ Use with a buddy ☐ Use at an OPS/SCS
☐ Other, specify______________________ ☐ Prefer not to say

23. If your drugs tested positive for benzodiazepines (before using), what would you do? (Select all that apply)
☐ Use less ☐ Use more slowly ☐ Would not use the drugs ☐ Continue using as usual
☐ Have someone check on me ☐ Use with a buddy ☐ Use at an OPS/SCS
☐ Other, specify______________________ ☐ Prefer not to say

24. Have you recently seen or heard an alert about recent drug overdoses, toxic drugs found e.g. from drug checking/testing, and other possible issues with street drugs? (Select one)
☐ Yes ☐ No *(skip to 25)* ☐ Prefer not to say *(skip to 25)*

a. **Where did you notice these alerts?** (Select all that apply)
☐ At harm reduction site/observed consumption site/community organization ☐ At healthcare provider
☐ On social media such as Facebook/Twitter ☐ Received an email/text ☐ On the news/media
☐ Heard about it from a friend/peer network ☐ Posters on the street
☐ Other, specify: ______________________ ☐ Prefer not to say

b. **Other than those listed above, where/how else do you think alerts should be posted/made available?**
Specify: ______________________

c. **Do you find these alerts useful?** (Select one)
☐ Yes. If so, why/how? ______________________
☐ No. If so, why not? ______________________ ☐ Prefer not to say

d. **Do you take any steps to be safer (get drugs checked/tested, use overdose prevention sites, use with a buddy, etc.) when you see an alert about drugs you may use?** (Select all that apply)
☐ Yes, I take precautions ☐ No, it doesn't change anything
☐ Other, specify ______________________ ☐ Prefer not to say

e. **Is there anything you can suggest to improve alerts?**
Specify: ______________________

25. Do you currently consider yourself to be at risk of experiencing an opioid overdose? (Select one)
☐ Yes ☐ No ☐ Don't know ☐ Prefer not to say

26. In the *last 6 months*, have YOU overdosed by accident from using opioids (e.g. fentanyl, heroin)? (Select one)
☐ Yes ☐ No *(skip to 27)* ☐ Don't know *(skip to 27)* ☐ Prefer not to say *(skip to 27)*

a. **In the *last 6 months*, how many times did you overdose by accident from using opioids?** (Select one)
☐ Once ☐ 2 times ☐ 3 times ☐ 4 times ☐ If 5 or more, specify: ______ ☐ Prefer not to say

b. **In the *last 6 months*, when you had the most recent opioid overdose, where did it happen?** (Select one)

☐ Private residence (including friends' and family's residence)
☐ Other residence(hotel/motel, rooming house, single room occupancy (SRO),social/supportive housing)
☐ Overdose prevention site (OPS)/supervised consumption site (SCS)
☐ Correctional facility/police cell ☐ Medical facility (hospital, clinic) ☐ Occupational site
☐ Public building (restaurants, businesses, public washrooms) ☐ Outside
☐ Other, specify: ____________ ☐ Prefer not to say

27. In the *last 6 months*, have YOU overdosed (overamped) by accident from using a stimulant (e.g. crystal meth, crack, cocaine)? (Select one)

☐ Yes ☐ No ☐ Don't know ☐ Prefer not to say

28. In the *last 6 months*, have you SEEN an accidental overdose in someone using any opioids? (Select one)

☐ Yes ☐ No ☐ Don't know ☐ Prefer not to say

29. Do you have a Naloxone/Narcan kit? (Select one)

☐ Yes ☐ No, I do not have a kit but I want one
☐ No, I don't have a kit and I don't want one ☐ Prefer not to say

30. In the *last 6 months*, did any of the following make it difficult for you to get a Naloxone/Narcan kit? (Select all that apply)

☐ Had no difficulties ☐ I don't need/want a kit ☐ I don't know where to get a kit
☐ Site was too far away ☐ Site not open when I needed it ☐ Site closed due to COVID-19 pandemic
☐ Worried about being exposed to COVID-19 at site ☐ Worried about being stigmatized
☐ Other, specify: ____________ ☐ Prefer not to say

31. In the *last 6 months*, how often did you pick up supplies (e.g. needles, meth pipes, condoms) from any site/outreach, either for yourself or another person? (Select one)

☐ Every day ☐ A few times a week ☐ A few times a month ☐ Once a month or less
☐ Never ☐ Prefer not to say

32. In the *last 6 months*, did any of the following make it difficult for you to pick up supplies (e.g. needles, meth pipes, condoms) from any site/outreach? (Select all that apply)

☐ Had no difficulties ☐ Site was too far away ☐ Site not open when I needed it
☐ Site closed due to COVID-19 pandemic ☐ Worried about being exposed to COVID-19
☐ Staff had negative attitude ☐ Concerned about confidentiality
☐ Site didn't have the supplies I needed, specify: ____________
☐ Other, specify: ____________ ☐ Prefer not to say

33. In the *last 6 months*, did any of the following make it difficult for you to dispose of used supplies at any site/outreach/drop box? (Select all that apply)

☐ Had no difficulties ☐ Did not know where to go ☐ Disposal box was full ☐ Disposal site was too far away
☐ Disposal site not open when I needed it ☐ Disposal site closed due to COVID-19 pandemic
☐ Worried about being exposed to COVID-19 ☐ Worried about being stigmatized
☐ Other, specify: ____________ ☐ Prefer not to say

34. In the *last 6 months*, have you injected any type of drug? (Select one)

☐ Yes ☐ No *(skip to 35)* ☐ Prefer not to say *(skip to 35)*

a. **In the *last 6 months*, did you have any trouble getting unused/sterile needles?** (Select one)

☐ Yes ☐ No ☐ Prefer not to say

b. **In the *last 6 months*, have you fixed with a needle that had been used by someone else?** (Select one)

☐ Yes ☐ No ☐ Prefer not to say

c. **What sort of filter do you usually use when you inject drugs?** (Select one)

☐ I don't use a filter ☐ Cotton filter (comes with cooker) ☐ Q-tip or cotton ball
☐ Sterifilt filter (filter with a green tip that clips onto the end of your needle) ☐ Cigarette filter
☐ Other, specify: ____________ ☐ Prefer not to say

35. In the *last 6 months*, have you smoked/inhaled any drugs other than cannabis or tobacco? (Select one)

☐ Yes ☐ No *(skip to 36)* ☐ Prefer not to say *(skip to 36)*

a. **In the *last 6 months*, have you used a glass pipe to smoke any drug?** (Select all that apply)

☐ Yes, straight pipe (stem) ☐ Yes, bowl pipe ☐ No, used foil to smoke *(skip to 36)*
☐ No, smoked with something else, specify: ________ *(skip to 36)* ☐ Prefer not to say *(skip to 36)*

b. **Where did you obtain your glass straight pipe or bowl pipe?** (Select one)

☐ Bought from harm reduction site ☐ Bought from store ☐ Bought from someone selling pipes
☐ Received one for free from a harm reduction site ☐ Borrowed one from a friend
☐ Made a homemade pipe ☐ Other, specify: ____________ ☐ Prefer not to say

c. **In the *past 6 months*, what did you do when you couldn't find a new (unused) glass pipe to smoke drugs?** (Select all that apply)

☐ I had no problems finding new (unused) pipes ☐ Injected instead ☐ Snorted/swallowed instead
☐ Waited until I found a new pipe ☐ Shared, borrowed, or bought a pipe used by someone else
☐ Cleaned a pipe I had ☐ Smoked without a pipe using (specify): ________
☐ Other, specify: ____________ ☐ Prefer not to say

36. In the *last 6 months*, have you used drugs at an overdose prevention site (OPS)/supervised consumption site (SCS)? (Select one)

An OPS/SCS is a place (fixed or mobile) where drug consumption is supervised by staff or volunteers to reduce overdose related deaths.

☐ Yes ☐ No *(skip to 37)* ☐ Prefer not to say *(skip to 37)*

a. **How often are you using an OPS/SCS?** (Select one)

☐ Every day ☐ A few times a week ☐ A few times a month
☐ Once a month or less ☐ Prefer not to say

b. **In the *last 6 months*, what type of OPS/SCS have you used?** (Select all that apply)

☐ Shelter or housing ☐ Community health centre/health clinic ☐ Stand-alone OPS/SCS facility
☐ Mobile Site ☐ Community Organization
☐ Other, specify: ______________________________ ☐ Prefer not to say

37. In the *last 6 months*, did any of the following make it difficult for you to use an OPS/SCS? (Select all that apply)

☐ Had no difficulties ☐ I don't need/want to use an OPS/SCS ☐ Service not available in my community
☐ Site too far away ☐ Site not open when I needed it ☐ Site closed due to COVID-19 pandemic
☐ Not allowed to smoke/snort drugs there ☐ Worried about being exposed to COVID-19
☐ Worried about being stigmatized ☐ Site was too busy/chaotic ☐ Staff had negative attitude
☐ Concerned about confidentiality ☐ Other, specify: ____________________ ☐ Prefer not to say

38. Have you experienced any of the following changes when buying street drugs since the COVID-19 pandemic began in March 2020? (Select all that apply)

☐ No, there has been no change in the drugs I buy ☐ The drugs I typically use are harder to find
☐ The drugs I typically use are more expensive ☐ The drugs I typically use are of lower quality
☐ The drugs I typically use are more toxic and/or potent
☐ Other, specify: ______________________ ☐ Prefer not to say

39. Have you used drugs on the street or in public more often since the COVID-19 pandemic began? (Select one)

☐ Yes ☐ No *(skip to 40)* ☐ Prefer not to say *(skip to 40)*

a. **If yes, why have you used drugs on the street or in public more often?** (Select all that apply)

☐ Couldn't access OPS/SCS ☐ Not allowed visitors at housing site ☐ Felt safer
☐ Did not want to use alone ☐ Other, specify: ________________ ☐ Prefer not to say

40. Do you receive income or disability assistance regularly? (Select one)

☐ Yes ☐ No *(skip to 41)* ☐ Prefer not to say *(skip to 41)*

a. **Since the COVID-19 pandemic began in March 2020, an additional $300 was added to monthly income and disability assistance payments. How did receiving the additional $300 each month affect you or help you?** (Select all that apply)

☐ It did not affect me ☐ It allowed me to follow physical distancing guidelines
☐ I engaged in less illegal/prohibited activities (e.g. sex work, panhandling) for money
☐ I was able to have better/healthier food ☐ I was able to pay my rent
☐ I was able to buy the drugs I needed to be well ☐ I was able to pay off some of my debt
☐ Other, specify: ________________________ ☐ Prefer not to say

41. Have you received Canada Emergency Response Benefit (CERB) or the Canada Recovery Benefit (CRB) since the COVID-19 pandemic began in March 2020? (Select one)

☐ Yes ☐ No *(skip to 42)* ☐ Prefer not to say *(skip to 42)*

a. **How did receiving CERB or CRB funds each month affect you or help you?** (Select all that apply)

☐ It did not affect me ☐ It allowed me to follow physical distancing guidelines
☐ I engaged in less illegal/prohibited activities (e.g. sex work, panhandling) for money
☐ I was able to receive compensation after losing my job ☐ I was able to pay my rent
☐ I was able to have better/healthier food
☐ I was able to buy the drugs I needed to be well ☐ I was able to pay off some of my debt
☐ Other, specify: ________________________ ☐ Prefer not to say

42. In the *last 6 months*, have you been reluctant to seek medical care because you use drugs? (Select one)

☐ Yes ☐ No ☐ Don't know ☐ Prefer not to say

43. In the *last 6 months*, have you been <u>denied</u> medical care you needed because you use drugs? (Select one)

☐ Yes ☐ No ☐ Don't know ☐ Prefer not to say

44. In the *last 6 months*, have you been <u>denied</u> housing because you use drugs? (Select one)

☐ Yes ☐ No ☐ Don't know ☐ Prefer not to say

45. In the *last 6 months*, have you been <u>denied</u> employment because you use drugs? (Select one)

☐ Yes ☐ No ☐ Don't know ☐ Prefer not to say

46. Have you heard of the overdose monitoring phone app, LifeGuard? (Select one)

☐ Yes ☐ No *(skip to 47)* ☐ Prefer not to say *(skip to 47)*

a. **Have you downloaded and used the LifeGuard app while using drugs?** (Select one)

☐ Yes *(skip to 47)* ☐ No ☐ Prefer not to say *(skip to 47)*

b. **Why have you not used the LifeGuard app?** (Select all that apply)

☐ I don't use opioids ☐ I don't have a phone ☐ I don't want to be monitored while using drugs
☐ I don't want emergency services to be alerted ☐ Concerned police will attend
☐ I never use alone ☐ The app is confusing
☐ Other specify: ______________________________ ☐ Prefer not to say

47. Have you heard of the overdose monitoring phone app, Be Safe (BRAVE)? (Select one)

☐ Yes ☐ No *(skip to 48)* ☐ Prefer not to say *(skip to 48)*

a. **Have you downloaded and used the Be Safe app while using drugs?** (Select one)

☐ Yes *(skip to 48)* ☐ No ☐ Prefer not to say *(skip to 48)*

b. **Why have you not used the Be Safe app?** (Select all that apply)

☐ I don't use opioids ☐ I don't have a phone ☐ I don't want to be monitored while using drugs
☐ I don't want emergency services to be alerted ☐ Concerned police will attend
☐ I never use alone ☐ The app is confusing
☐ Other specify: ______________________________ ☐ Prefer not to say

48. Have you heard about the Good Samaritan Drug Overdose Act? (Select one)

☐ Yes ☐ No ☐ Prefer not to say

THANK YOU FOR TAKING THE SURVEY!
